# Systemic inflammatory regulators and proliferative diabetic retinopathy: A bidirectional Mendelian randomization study

**DOI:** 10.3389/fimmu.2023.1088778

**Published:** 2023-02-10

**Authors:** Qiqin Shi, Qiangsheng Wang, Zhenqian Wang, Jiawen Lu, Ruobing Wang

**Affiliations:** ^1^ Department of Ophthalmology, Ningbo Hangzhou Bay Hospital, Ningbo, Zhejiang, China; ^2^ Department of Haematology, Ningbo Hangzhou Bay Hospital, Ningbo, Zhejiang, China; ^3^ School of Public Health (Shenzhen), Sun Yat-sen University, Shenzhen, Guangdong, China; ^4^ Department of Ophthalmology, Renji Hospital, School of Medicine, Shanghai Jiao Tong University, Shanghai, China

**Keywords:** bidirectional, systemic inflammatory regulators, Mendelian randomization, proliferative diabetic retinopathy, meta-analysis

## Abstract

**Background:**

Increasing evidence shows that systemic inflammation is an embedded mechanism of proliferative diabetic retinopathy (PDR). However, the specific systemic inflammatory factors involved in this process remained obscure. The study aimed to identify the upstream and downstream systemic regulators of PDR by using Mendelian randomization (MR) analyses.

**Methods:**

We performed a bidirectional two-sample MR analysis implementing the results from genome-wide association studies for 41 serum cytokines from 8,293 Finnish individuals, and PDR from FinnGen consortium (2,025 cases vs. 284,826 controls) and eight cohorts of European ancestry (398 cases vs. 2,848 controls), respectively. The inverse-variance-weighted method was adopted as the main MR method, and four additional MR methods (MR-Egger, weighted-median, MR-pleiotropy residual sum and outlier (MR-PRESSO), and MR-Steiger filtering methods) were used for the sensitivity analyses. Results from FinnGen and eight cohorts were pooled into a meta-analysis.

**Results:**

Our results showed that genetically predicted higher stem cell growth factor-β (SCGFb) and interleukin-8 were positively associated with an elevated risk of PDR, with a combined effect of one standard deviation (SD) increase in SCGFb and interleukin-8 causing 11.8% [95% confidence interval (CI): 0.6%, 24.2%]) and 21.4% [95% CI: 3.8%, 41.9%]) higher risk of PDR, respectively. In contrast, genetically predisposition to PDR showed a positive association with the increased levels of growth-regulated oncogene-α (GROa), stromal cell-derived factor-1 alpha (SDF1a), monocyte chemotactic protein-3 (MCP3), granulocyte colony-stimulating factor (GCSF), interleukin-12p70, and interleukin-2 receptor subunit alpha (IL-2ra).

**Conclusions:**

Our MR study identified two upstream regulators and six downstream effectors of PDR, providing opportunities for new therapeutic exploitation of PDR onset. Nonetheless, these nominal associations of systemic inflammatory regulators and PDR require validation in larger cohorts.

## Introduction

Diabetic retinopathy (DR) is a specific microvascular complication of diabetes mellitus and is the leading cause of preventable blindness in the adult working population (1). The damage of DR starts with non-proliferative diabetic retinopathy (NPDR) and progresses to an advanced stage as proliferative diabetic retinopathy (PDR) (2). PDR is a quite severe condition that can result in the progressive loss of peripheral and central vision in patients (3). Throughout the period 1980 to 2008, 35 studies conducted worldwide estimated the global prevalence of DR and PDR at 35.4% and 7.5%, respectively (4). Clinically, PDR is commonly diagnosed by fluorescein angiography using a contrast agent which may cause nausea in 10% of patients and even anaphylaxis or death (5). Thus, it is important and necessary to explore noninvasive, reliable, and practical biomarkers for preoperatively predicting the risk of PDR.

Increasing evidence shows that systemic inflammation is an intrinsic mechanism of occurrence and development of DR (6). Normally, the retina is an immune-privileged region that is divided by blood-retinal barriers that restricts exchange of proteins and fluids. However, long-term exposure to chronic inflammation can cause the destruction of the blood-retina barrier, triggering bleeding, exudation, edema of the retinal tissue, which mark the onset of DR (7, 8). With further endothelial cell damage and abnormal capillary basement membrane function, the disease progresses to PDR and the automatic regulation of capillary blood flow is severely interrupted (9). In this process, a critical cause and trigger is ischemia-induced angiogenesis, inflammation, and fibrosis of the retina (10). Research in recent years has emphasized the role of the vascular endothelial growth factor (VEGF) and other bioactive substances with angiogenic and antiangiogenic activity in the pathogenesis of PDR (such as platelet-derived growth factor (PDGF), interleukin 8 (IL-8), monocytes Chemotactic protein 1 (MCP)-1), tumour necrosis factor-α (TNF-α)) (11, 12). Despite promising results from clinical trials of novel of new treatments targeting these factors, few have reached significant and long-term clinical success. In one example, clinical studies have shown that widespread anti-VEGF agents do not target the underlying DR pathogenesis, therefore cannot resolve DR onset and progression (13). For that, one can speculate that the same factor may exert distinct roles in different cell types and different developmental stages, which makes it difficult to determine its specific role in the PDR onset. For instance, the role of IL-18 in PDR remains enigmatic as it can either promote or suppress angiogenesis (14). Moreover, most previous studies exclusively focused on the roles of some specific inflammatory factors in vitreous or intraocular fluid while the systemic circulating changes of inflammatory factors are equally important (15). Finally, considering observational studies are vulnerable to bias and reverse causation, they are not appropriate for identifying the upstream and downstream regulatory mechanisms of PDR.

Mendelian randomization (MR) is an analytic approach to establishing causality between a gene product and intermediate phenotype which is in essence tantamount to a randomized clinical trial (16). It is designed based on the fact that genetic variants are randomly allocated during gamete formation and conception, therefore, are free of reverse caution and confounding bias (17). By using MR methods, Han et al. identified a strong causal relationship between circulating C-reactive protein levels and age-related macular degeneration which provided new insights into the role of systemic therapies (18). Furthermore, a bidirectional MR analysis, as an extension of the traditional MR method, could help tease apart complex relationships of biological systems, such as the existence of feedback loops between exposure and outcome variables (19). For example, a bidirectional MR study also found that targeted interventions of several specific inflammatory factors could alleviate the risk of multiple myeloma (20). Herein, we took advantage of the largest publicly available genome-wide association study (GWAS) data available for human cytokines and PDR to identify the upstream regulators and downstream effectors for PDR using a two-sample bidirectional MR.

## Methods

### Study design

**Figure 1:**
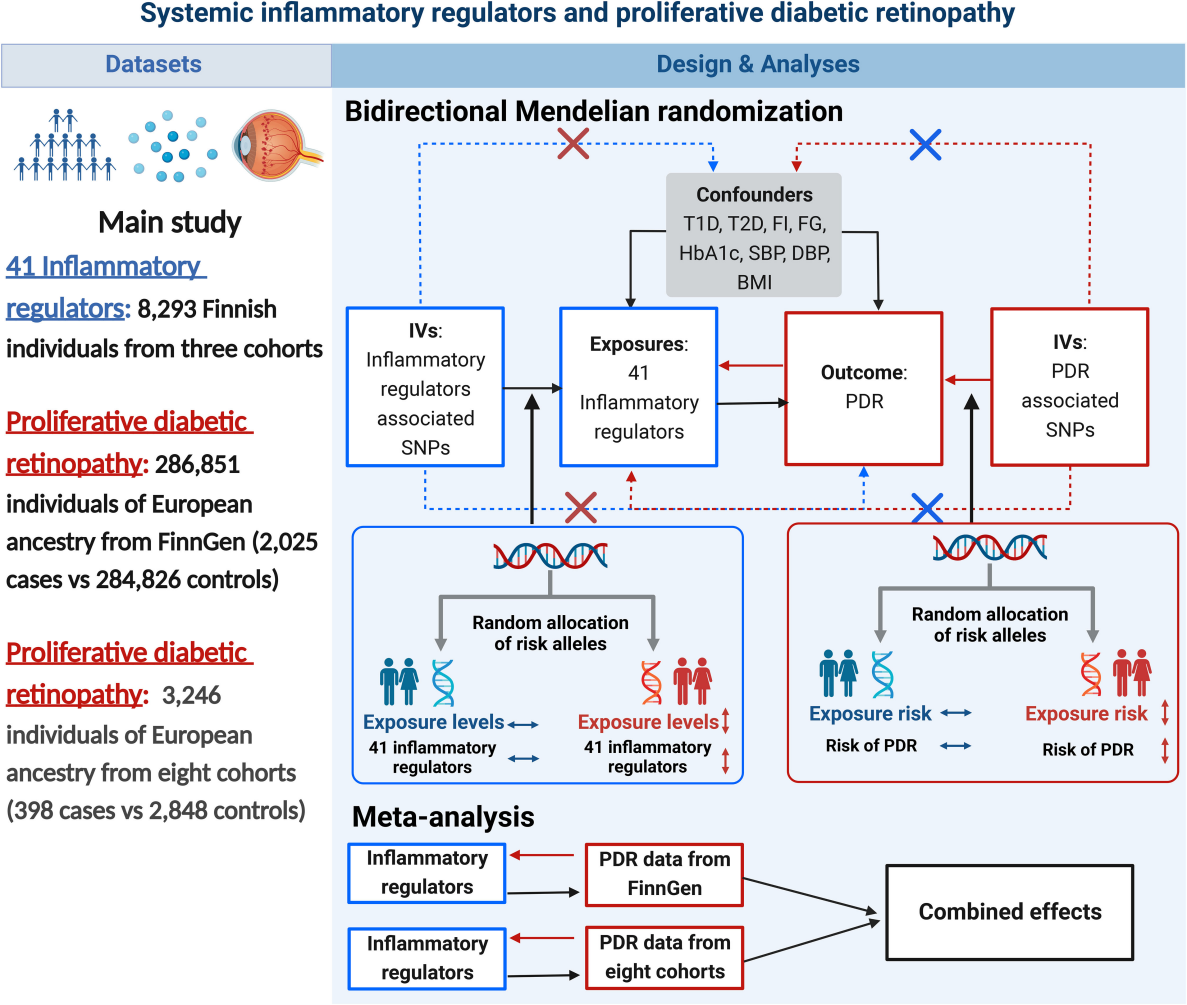
Datasets, assumptions, and study design of the bidirectional Mendelian randomization study of the associations between 41 inflammatory factors and PDR. BMI, body mass index; DBP, diastolic blood pressure; FG, fasting glucose; FI, fasting insulin; IVs, instrumental variables; PDR, proliferative diabetic retinopathy; SBP, systolic blood pressure; SNPs, single-nucleotide polymorphisms; T1D, type 1 diabetes; T2D, type 2 diabetes.

A brief description of the bidirectional MR design is displayed in **Figure 1**. Genetic instruments for 41 systemic inflammatory regulators were obtained from published GWAS of three Finnish cohorts (21). Data for PDR were obtained from FinnGen (22) and eight cohorts of European ancestry (23). First, we selected genetic variants for each inflammatory regulator to infer the causality from each inflammatory regulator to PDR. Second, genetic variants associated with PDR were exploited to infer the causality from PDR to inflammatory regulators. Finally, we combined estimates from two sources of PDR using meta-analysis method. Studies included in the original GWASs had been approved by a relevant institutional review board.

### Data source for inflammatory regulators

The published large-scale GWAS meta-analysis for the circulating concentrations of 41 cytokines was used in this study, which included up to 8,293 Finnish individuals from three independent population cohorts: the Cardiovascular Risk in Young Finns Study (YFS), FINRISK1997, and FINRISK2002 (21). The quantification of cytokines was performed from EDTA plasma in FINRISK 1997, from heparin plasma in FINRISK 2002, and from serum in YFS and measured by Bio-Rad’s premixed Bio-Plex Pro Human Cytokine 27-plex Assay and 21-plex Assay, and a Bio-Plex 200 reader with Bio-Plex 6.0 software. Those 41 cytokines distributions were normalized with two-step inverse transformation. To be specific, we first normalized cytokine distributions by inverse transformation, and then performed inverse transformation for residuals of linear regression model of transformed cytokines on age, sex, body mass index (BMI), and genetic principal components. Genotype imputation was performed based on reference haplotypes provided by the 1000 Genomes Project Phase 1. In each cohort, an additive model with adjustment for age, sex, BMI and the first 10 genetic principal components were performed to test genetic associations between 10.7 million SNPs and 41 cytokine concentrations. Meta-analyses were performed to combined genetic associations across the three cohorts (21).

### Data sources for PDR

Summary-level data on PDR was extracted from a GWAS of 286,851 individuals of European ancestry from the FinnGen consortium (R7 release) (22). The FinnGen Study is a Finnish, nationwide GWAS meta-analysis of 9 biobanks, which has very limited overlap (<3%) with the GWAS of inflammatory regulators. Thus, we considered the risk of bias due to sample overlap minimal (24). The biobanks have been linked with longitudinal digital health record data from nationwide health registries. The GWAS of PDR in the FinnGen Study included 2,025 PDR cases and 284,826 controls. The PDR was defined as the later stage of diabetic retinopathy, characterized by neovascularisation of the retina in ICD-10 (code: H36.03). The genetic associations were adjusted for age, sex, 10 principal components, and genotyping batch. More details in FinnGen were described in https://finngen.gitbook.io/documentation/v/r7/.

Another PDR summary-level data derived from GWAS of eight European cohorts (23). Early Treatment Diabetic Retinopathy Study (ETDRS) score was used to define PDR case in the GWAS study (25). GWAS meta-analysis of PDR included 398 individuals (ETDRS ≥60) with PDR and 2,848 controls. Liability threshold modelling of the duration of diabetes and glycaemic control and five principal components were considered in this GWAS meta-analysis (23). The samples of this study were non-overlapped with the samples of cytokines GWAS.

### Selection of genetic instrumental variables

To satisfy the MR assumptions (**Figure 1**), all SNPs are strongly and independently (R^2^ < 0.001 within 10 Mb) predicted exposures from the published GWAS at genome-wide significance (*P <*5×10^-8^). Since only 8 systemic inflammatory regulators had 3 or more independent SNPs that reached genome-wide significance, we adopted less stringent thresholds of 5×10^-6^ to obtain more SNPs for inflammatory regulators. The thresholds are appropriate to select genetic instrumental variables as described before (26).

A critical assumption of the MR design is that SNPs should influence the outcome only through the exposure of interest. Using the publicly available GWAS summary data, we examined whether any of these SNPs were associated with common confounders (T1D, T2D, HbA1c, SBP, and DBP, fasting glucose, fasting insulin, and BMI) and outcomes at a *P*-value of Bonferroni level (0.05/number of SNPs). For T1D, the genetic associations were derived from a meta-analysis of GWASs in 25,063 (9,358 cases and 15,705 controls) European-descent individuals (27). The genetic associations of these SNPs with T2D were obtained from a meta-analysis of GWASs in 898,130 European-descent individuals (9% cases) (28). For HbA1c, fasting glucose, and fasting insulin, the genetic associations were derived from a GWAS meta-analysis in ~200,000 European-ancestry individuals without diabetes (29). For blood pressure, the associations of these SNPs with SBP or DBP were obtained from a meta-analysis of GWAS in 757,601 individuals of European ancestry, which included 458,577 participants from UK Biobank and 299,024 participants from international consortium for blood pressure (ICBP) (30). The associations of these SNPs with inverse-normally transformed BMI were obtained from a meta-analysis of GWASs in ~700 000 participants of European ancestry (31).

At last, we quantified the strength of SNPs using the mean *F*-statistic (32). Mean *F-*statistic >10 suggested sufficient strength to ensure the validity of the SNPs for the trait.

### Statistical analysis

We performed a bidirectional two-sample MR method using summary association data to explore the causal direction of relationship between inflammatory regulators and PDR. If the SNP associated with the exposure is missing from the outcome GWAS, we replaced the SNP by a proxy SNP in high linkage disequilibrium (R^2^ >0.80) with the SNP using LDlink (https://ldlink.nci.nih.gov/) (33).

We critically performed data harmonization to make sure that the effect of a SNP on the exposure and the outcome corresponded to the same allele. For SNPs with different effect alleles due to different strands, we corrected the strand and ensured same effect allele in both datasets. However, palindromic SNPs are much harder to harmonize because the alleles are the same on both strands, then we deleted them to avoid ambiguity as to whether exposure and outcome GWAS report the same effect allele (34). In the main analysis, we calculated a Wald ratio estimate for each genetic variant and summarized the estimates using the inverse-variance weighted (IVW) method. The IVW with multiplicative random effects method provides a concise estimation and takes into account potential heterogeneity among the Wald ratio estimates from SNPs (35). Thus, if there is heterogeneity, random-effects IVW models are applied; otherwise, the fixed-effect IVW model is applied. Estimates of bidirectional associations of inflammatory regulators with the risk of PDR were from a combination of FinnGen consortium data and data of eight cohorts using the meta-analysis. Heterogeneity of meta-analysis was examined by the Cochran *Q* test and *I* ^2^. Scatter plots depicting the bidirectional causal associations of systemic inflammatory regulators with PDR were also provided. The effects in 41 cytokines were reported as changes in inverse normalized cytokines concentration per effect allele dosage. Results of the effect of 41 cytokines on PDR are presented as ORs (95% CIs) per 1 SD genetic predicted cytokine change. The effects of PDR on systemic inflammatory regulators were reported as *β* coefficients and 95% CIs.

### Sensitivity analysis

We performed a set of sensitivity analyses using methods with different assumptions about horizontal pleiotropy, including MR-Egger, weighted median, and Mendelian Randomization Pleiotropy RESidual Sum and Outlier (MR-PRESSO). MR-Egger analysis provides an assessment of instrumental variable pleiotropy, with a non-zero intercept indicating that the IVW estimate is biased (36). The weighted median can provide a consistent estimate for the causal effect even if up to half of the SNPs violate horizontal pleiotropy (37). MR-PRESSO uses the global test to detect horizontal pleiotropy, and if necessary, could correct for potentially pleiotropic outliers *via* outliers removal (38). Heterogeneity in the IVW estimates was examined by the Cochran Q test and *I* ^2^ index. We further repeated the analyses after MR-Steiger filtering which removes SNPs suggestive of a reversed causal direction. MR-Steiger filtering assumes a valid genetic variant should explain more variance in the exposure than the outcome and genetic variant are identified to have bidirectional effects if genetic instruments do not satisfy this criterion (39).

The Bonferroni method was used to correct for multiple testing and, therefore, we considered associations with *P*-values below 0.0012 (0.05/41) as strong evidence of associations. Results with *P*-values between 0.0012 and 0.05 were regarded as suggestive associations. All analyses were two-sided and conducted using TwoSampleMR (version 0.5.6) and MRPRESSO (version 1.0) packages in R software (version 3.6.3). Reporting follows the STROBE-MR statement.

## Results

### Causal effects of inflammatory regulators on PDR

**Figure 2:**
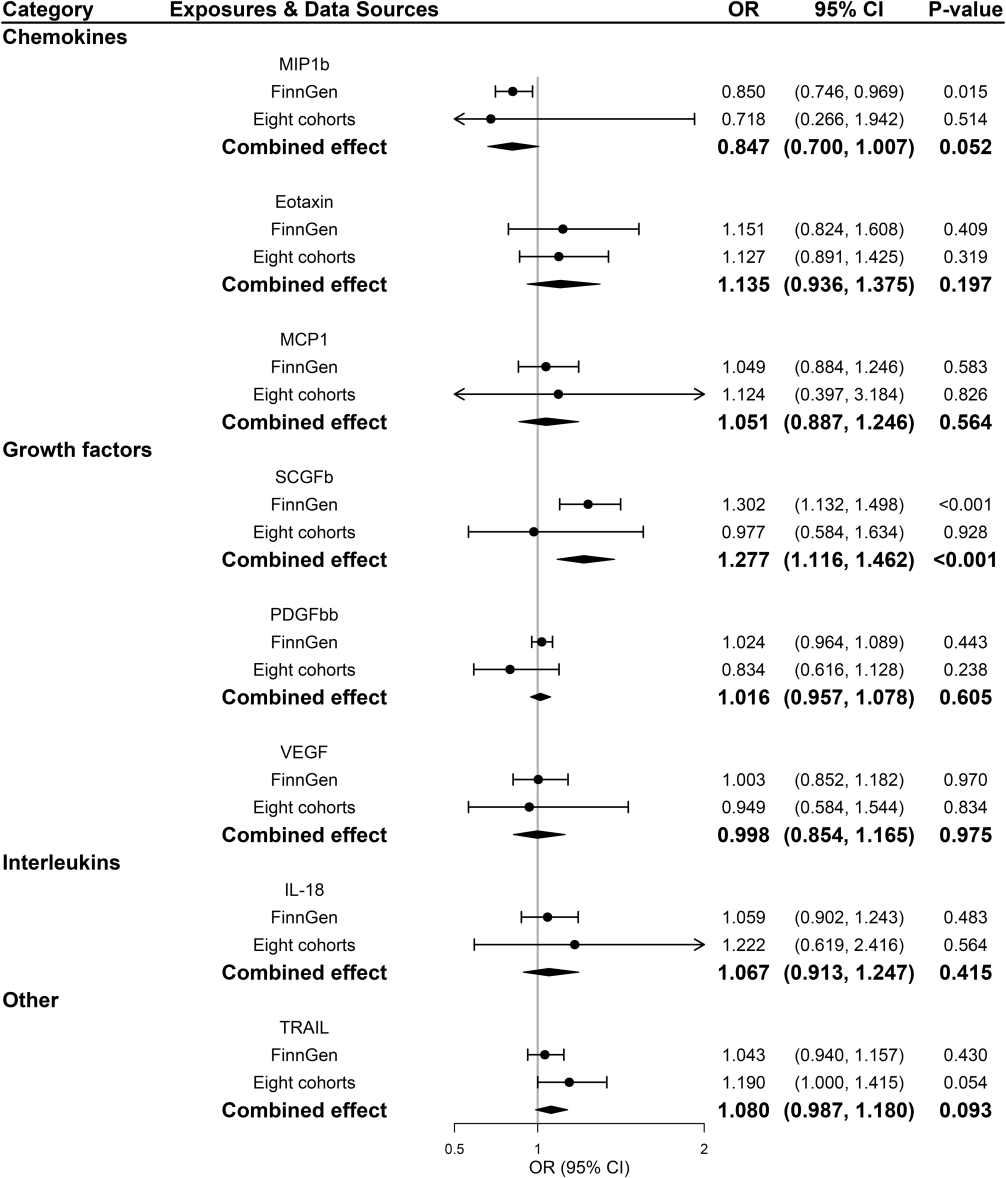
Associations between genetically predicted systemic inflammatory regulators and proliferative diabetic retinopathy (with genome-wide significant SNPs). IL, interleukin; MCP1, monocyte chemotactic protein-1; MIP1b, macrophage inflammatory protein-1 beta; PDGFbb, platelet-derived growth factor BB; SCGFb, stem cell growth factor beta; SNPs, single-nucleotide polymorphisms; TRAIL, TNF-related apoptosis-inducing ligand; VEGF, vascular endothelial growth factor.

Across 8 inflammatory regulators that we examined, estimates of *F*-statistics for their respective SNPs with *P*-value <5×10^-8^ ranged from 202.48 to 1053.06, suggesting that weak instruments bias would be minimal (**Supplementary Tables 1**, **3**). **Figure 2** showed causal relationships between the 8 systemic inflammatory regulators and PDR risk with SNPs reaching *P* < 5 × 10^-8^. Among them, genetically predicted higher stem cell growth factor *β* (SCGFb) was strongly associated with elevated risk of PDR in both FinnGen consortium data and meta-analysis (*P*<0.001), with a combined OR of one SD increase in genetically predicted SCGFb causing 27.7% ([95% CI: 11.6%,46.2%]; *P*<0.001) higher risk of PDR. Additionally, there was no evidence of other 7 inflammatory regulators associated with PDR in meta-analysis (all *P >*0.05). Weighted median method gave consistent results and MR-Egger suggested no evidence of horizontal pleiotropy in the two data sources (**Supplementary Tables 2**, **4**). MR-Steiger filtering detected no invalid genetic instruments for these analyses. No evidence of heterogeneity was detected by Cochran Q test (all *P*-values for Cochran Q test>0.05), thus, fixed-effects meta-analyses were applied in the 8 inflammatory regulators (**Supplementary Table 5**).

**Figure 3:**
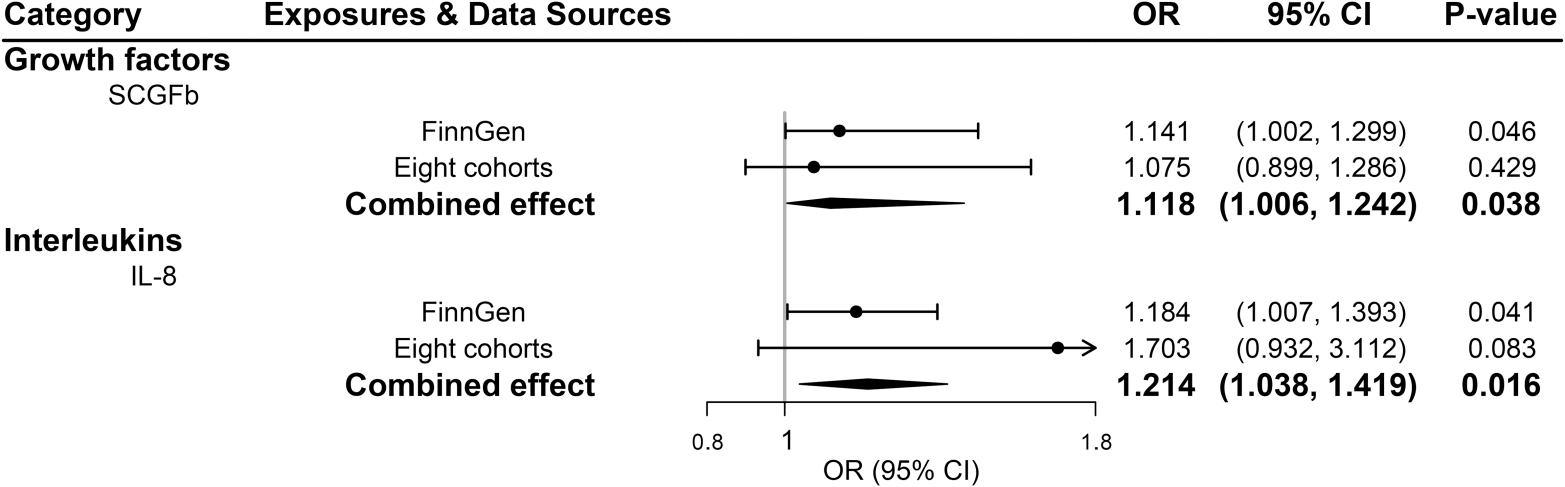
Significant associations between genetically predicted systemic inflammatory regulators and proliferative diabetic retinopathy (with SNPs reaching *P*<5×10^-6^). IL, interleukin; SCGFb, stem cell growth factor beta.

**Figure 4:**
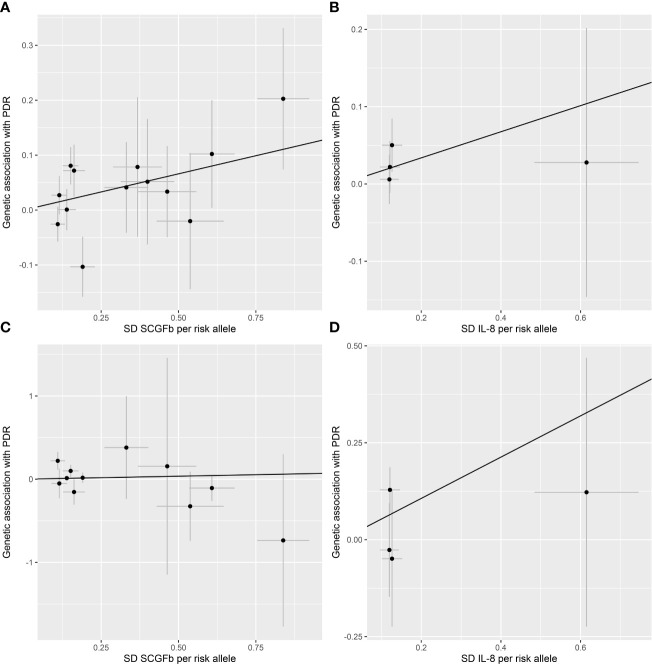
Scatter plot of SCGFb and IL-8 related SNPs with the risk of proliferative diabetic retinopathy. **(A)** Genetic association of SCGFb related SNPs and PDR from FinnGen. **(B)** Genetic association of IL-8 related SNPs and PDR from FinnGen. **(C)** Genetic association of SCGFb related SNPs and PDR from eight cohorts. **(D)** Genetic association of IL-8 related SNPs and PDR from eight cohorts. Black line indicates the estimate of effect using the inverse variance weighted method. Circles indicate marginal genetic associations with SCGFb or IL-8 and risk of outcome for each variant. Error bars indicate 95% confidence intervals. IL, interleukin; SCGFb, stem cell growth factor beta; SD, standard deviation.

All 41 inflammatory regulators using the less stringent cut-off of *P <*5×10^-6^ had 3 or more SNPs with the *F*-statistics ranged from 80.79 to 1923.92 in two data sources (**Supplementary Tables 6**, **8**). The primary IVW method showed genetically determined levels of SCGFb and IL-8 were suggestively positive associated with the risk of PDR in FinnGen consortium and meta-analysis (**Figure 3**). The combined ORs of PDR were 1.118 [95% CI:1.006, 1.242] per a 1-SD increase in SCGFb and 1.214 [1.038, 1.419] per a 1-SD increase in IL-8 (**Figure 3**). Associations between each instrumental variable of SCGFb and risk of PDR are shown in **Figures 4A, C** for summary-level data on PDR from FinnGen and eight cohorts, respectively. Similarly, **Figures 4B, D** showed the associations between IL-8 and PDR from FinnGen and eight cohorts, respectively. No other associations were observed in the study (**Supplementary Tables 7**, **9**, **10**). MR-Egger intercept test yielded no indication of potential pleiotropy in both datasets (all *P*-value >0.05; **Supplementary Tables 7**, **9**), while MR-PRESSO suggested pleiotropic SNPs may present for monocyte chemotactic protein-1 (MCP1), monokine induced by interferon gamma (MIG), and macrophage colony-stimulating factor (MCSF) in FinnGen consortium data but the results do not alter after removing outliers (**Supplementary Table 7**). No more significant effects were detected in the weighted median method and MR-Egger method (**Supplementary Tables 7**, **9**). No invalid SNPs were detected by MR-Steiger filtering method. Since there is evidence of heterogeneity for basic fibroblast growth factor (FGFBasic) in meta-analysis, random-effects methods were performed to combine effects for FGFBasic (**Supplementary Table 10**).

### Causal effects of PDR on inflammatory regulators

**Figure 5:**
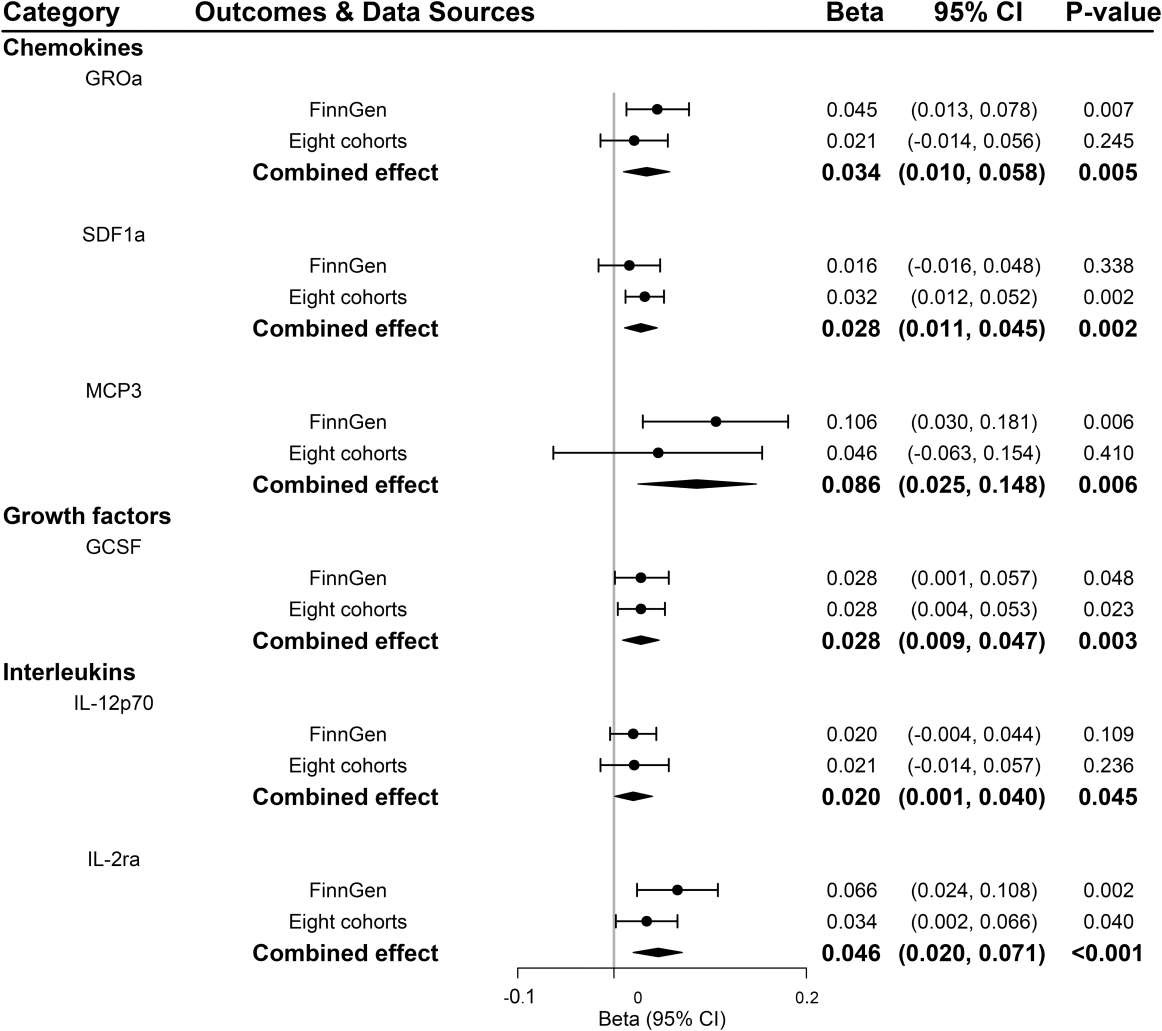
Significant associations between genetically predicted proliferative diabetic retinopathy and systemic inflammatory regulators. GCSF, granulocyte colony-stimulating factor; GROa, growth-regulated oncogene-alpha; IL, interleukin; MCP3, monocyte chemotactic protein-3; SDF1a, stromal cell-derived factor 1.

According to the process of SNPs selection mentioned in the method, we extracted 20 SNPs reaching a genome-wide significance *P*-value threshold of 5×10^-8^ for PDR in the FinnGen consortium, while a set of 10 SNPs reported to be associated with PDR at a less stringent *P*-value threshold of 5×10^-6^ in the GWAS of eight cohorts. The *F*-statistics for FinnGen consortium data and data of eight cohorts were 1195.91 and 240.73, respectively, suggesting that weak instrument bias was minimal (**Supplementary Tables 11**, **13**). We observed higher genetically predicted IL-2ra and GCSF that were consistent in FinnGen consortium data, eight cohorts and meta-analysis (all *P*<0.05; **Figure 5**). Specifically, one-unit increase in log OR of PDR strongly leading to 0.046 [95% CI: 0.020-0.071] SD higher levels of IL-2ra (*P <*0.001) and 0.028 [95% CI: 0.009-0.047] SD higher levels of GCSF (*P* =0.003) in meta-analysis (**Figure 5**). In addition, genetic predisposition to PDR showed suggestively positive association with the increased levels of stromal cell-derived factor-1 alpha (SDF1a), monocyte chemotactic protein-3 (MCP3), and IL-12p70 in meta-analyses (all *P*<0.05; **Figure 5**). Scatter plots of relationships between genetically predicted PDR and these inflammatory regulators were provided in **Supplementary Figure S1** for FinnGen and **Supplementary Figure S2** for eight cohorts. There was no evidence for causal associations between PDR and other inflammatory regulators (**Supplementary Table 15**). The MR-Egger intercept detected no horizontal pleiotropy, while MR-PRESSO global test for MIP1b, MIG and CTACK in FinnGen consortium data and FGFBasic in data of eight cohorts suggested that there may be pleiotropic SNPs (*P* for global test <0.05) but the MR-PRESSO outlier-adjusted causal estimates did not alter the results (**Supplementary Tables 12**, **14**). No invalid SNPs were detected by MR-Steiger filtering method.

## Discussion

To our knowledge, this is the first study that comprehensively evaluated the causal effects of 41 systemic inflammatory regulators on PDR, and vice versa. We performed a two-sample bidirectional MR analysis in two independent populations and combined the results to a meta-analysis. Our results suggested that genetically predicted SCGFb and IL-8 were positively associated PDR risk while genetic predisposition to PDR suggestively contributed to an increase in GROa, SDF1a, MCP3, GCSF, IL2-ra and IL-12p70. These findings were generally robust in sensitivity analyses.

Previous studies demonstrated that inflammation plays a pivotal molecular basis in the pathogenesis of DR (40). Several inflammatory molecules, like IL-6, IL-8, IL-10, TNF-a, and VEGF, have been proposed as serum biomarkers of PDR (41). However, these observational studies could be subjected to confounding factors and reverse caution, distorting the true causal relationships. In this study, we performed a two-sample bidirectional MR analysis and identified the upstream and downstream inflammatory regulators of PDR. Generally consistent with prior studies, our results identified that elevated IL-8 and SCGFb levels are associated with increased risk of PDR. IL-8 was a well-established pro-inflammatory cytokine and plays an essential role in initiating and strengthening inflammatory cascade (42). Meanwhile, it is one of the most consistently reported up-regulated cytokines in DR patients (42–44). Numerous studies have detected higher levels of IL-8 in PDR patients, leading to the inference that IL-8 may have a synergistic effect on the pathogenesis of the disease (43, 44). Biologically, IL-8 belongs to a family of ELR+ CXC chemokines with the ELR motif that mainly attract neutrophils (45). It exerts its effects on neutrophils *via* two different cell surface receptors initially named as CXCR1 and CXCR2, mediating and regulating leukocyte recruitment and activation at sites of inflammation (46), followed by ischemia, promoting leakage, and neovascularization (47). Within the eye, IL-8 can likewise promote angiogenesis, induce superoxide and peroxide production in endothelial cell, thereby destroying the blood-eye barrier and finally leading to PDR (48). Second, SCGFb is a newly found protein secreted sulfated glycoprotein and functions as a growth factor at the early stage of hematopoiesis (49). It is selectively produced by bone and hematopoietic stromal cells and can mediate their proliferative activity against primitive hematopoietic progenitors (50). One recent case-control study suggested that SCGFb showed significantly higher expression levels in proliferative vitreoretinopathy, although no difference between PDR and other eye disease types (51). Therefore, SCGFb may have the potential to promote the PDR onset, but mechanisms behind this remain to be elucidated. Nonetheless, novel biomarkers also can complement limitations of traditional biomarkers in routine clinical practice.

In PDR stage, new aberrant blood vessels are fragile and could lead to vitreous hemorrhage and/or tractional retinal detachment from progressive fibrosis (52). In response to these injury, some retinal cell types, such as astrocytes and neurons, can upregulate the expression of various gene encoding cytokines, chemokines and elements of the complement cascade, promoting retinal degeneration (53). As expected, we observed a group of genetically increased systemic inflammatory regulators levels derived from PDR. Some of them were previously reported to be related to PDR, such as GROa, SDF1a, GCSF (54–57), while other inflammatory factors, as MCP-3, IL-12p70 and IL-2ra have been rarely studied so far. From limited available data, MCP-3, was only investigated in a gene array analysis based on an animal model study and found to be significantly upregulated among all member of the chemokine family (58). As for IL-2ra, a study provided clues into the underlying the causality between IL-2ra and PDR as the elevated plasma levels of soluble IL-2ra was positively with the vascular complications (59). Besides, limited information is available regarding the roles of IL-12p70 due to its low activity.

The study has several strengths. First, this is the first MR study to evaluated the causal relationship between systemic inflammatory regulators and PDR using an up-to-date summary-level data. Traditional observational studies are prone to be biased by reverse causality, as diabetes-induced hyperglycemia could cause retinal micro vasculopathy, inflammation, and retinal neurodegeneration as well. In this bidirectional MR study, we were able to avoid reverse causality and minimize residual confounding. Second, we conducted a meta-analysis by combining results from two data sources of PDR to increase statistical power, which guaranteed the robustness of our findings. Third, in a clinical practice, serum is one of the most accessible and easily obtained biofluids which allows for sample collection from both DR patients and healthy controls, whereas vitreous and aqueous sample collection requires highly invasive procedures for DR patients. Furthermore, previous research has been mainly based on the pathogenic mechanism involved in the development of PDR but our study focuses on both upstream and downstream circulating biomarkers that could be responsible for the prediction or treatment of PDR.

However, the study had several limitations. First, not all relevant SNPs of exposure availably obtained in the outcome GWAS, even after searching for potential proxies. Despite affecting the statistical power to detect small effects, we could still include a fair number of SNPs and perform adequate MR analyses. Second, although genetic instruments for inflammatory regulators were extracted from the largest current GWAS source of inflammatory regulators, there were still only a few genome-wide significant SNPs available for eight inflammatory regulators. A relative relaxed threshold of 5×10^-6^ was adopted for selecting instruments, which was considered as rational threshold (26). Moreover, the *F*-statistics of inflammatory were all greater than 10, suggesting the weak instruments bias is minimal. Third, although we cannot rule out the possibility of pleiotropy, we have excluded SNPs associated with potential confounders and conducted multiple sensitivity analyses (e.g., MR-Egger and MR-PRESSO) with different assumptions, which showed similar conclusions. Forth, MR is not perfectly analogous to a randomized controlled trial (RCT). Therefore, causal relationships of systemic inflammatory cytokines with PDR derived from MR analyses may differ in magnitude from those anticipated in an RCT and should be explained as life-course effects. Finally, our study only included participants of European descent, which could limit the generalizability of our results to other ethnicities.

## Conclusion

The present bidirectional MR study identified two upstream regulators and six downstream effectors of PDR. These findings provided opportunities for new therapeutic exploitation of PDR onset and permit us to implement a more personalized treatment with better visual function outcomes. Additional research is warranted to validate the role of specific inflammatory regulators on PDR.

## Data availability statement

The original contributions presented in the study are included in the article/**Supplementary Material**, further inquiries can be directed to the corresponding author/s.

## Author contributions

QS: Conceptualization; Data curation; Formal analysis; Investigation; Methodology; Software; Visualization; Roles/Writing - original draft; Writing - review & editing. QW: Conceptualization; Formal analysis; Funding acquisition; Investigation; Methodology; Roles/Writing - original draft; Writing - review & editing. ZW: Data curation; Formal analysis; Software; Validation; Writing - review & editing. JL: Visualization; Validation; Supervision; Writing - review & editing. RW: Conceptualization; Investigation; Methodology; Project administration; Visualization; Supervision; Writing - review & editing. All authors contributed to the article and approved the submitted version.

## References

[1] Saeedi P Petersohn I Salpea P Malanda B Karuranga S Unwin N. Global and regional diabetes prevalence estimates for 2019 and projections for 2030 and 2045: Results from the international diabetes federation diabetes atlas, 9 edition. Diabetes Res Clin Pract (2019) 157:107843. doi: 10.1016/j.diabres.2019.107843 31518657

[2] Nawaz IM Rezzola S Cancarini A Russo A Costagliola C Semeraro F. Human vitreous in proliferative diabetic retinopathy: Characterization and translational implications. Prog Retin Eye Res (2019) 72:100756. doi: 10.1016/j.preteyeres.2019.03.002 30951889

[3] Chaudhary S Zaveri J Becker N. Proliferative diabetic retinopathy (PDR). Disease-a-month DM (2021) 67(5):101140. doi: 10.1016/j.disamonth.2021.101140 33546872

[4] Solomon SD Chew E Duh EJ Sobrin L Sun JK VanderBeek BL. Diabetic retinopathy: A position statement by the American diabetes association. Diabetes Care (2017) 40(3):412–8. doi: 10.2337/dc16-2641 PMC5402875 28223445

[5] Wang M Garg I Miller JB. Wide field swept source optical coherence tomography angiography for the evaluation of proliferative diabetic retinopathy and associated lesions: A review. Semin Ophthalmol (2021) 36(4):162–7. doi: 10.1080/08820538.2021.1887901 33734945

[6] Tang J Kern TS. Inflammation in diabetic retinopathy. Prog In Retinal Eye Res (2011) 30(5):343–58. doi: 10.1016/j.preteyeres.2011.05.002 PMC3433044 21635964

[7] Campbell M Humphries P. The blood-retina barrier: tight junctions and barrier modulation. Adv In Exp Med Biol (2012) 763:70–84. doi: 10.1007/978-1-4614-4711-5_3 23397619

[8] Sigurdardottir S Zapadka TE Lindstrom SI Liu H Taylor BE Lee CA. Diabetes-mediated IL-17A enhances retinal inflammation, oxidative stress, and vascular permeability. Cell Immunol (2019) 341:103921. doi: 10.1016/j.cellimm.2019.04.009 31076079 PMC6599623

[9] Mammadzada P Bayle J Gudmundsson J Kvanta A André H. Identification of diagnostic and prognostic microRNAs for recurrent vitreous hemorrhage in patients with proliferative diabetic retinopathy. J Clin Med (2019) 8(12):2217. doi: 10.3390/jcm8122217 PMC6947310 31847440

[10] Abu El-Asrar AM Nawaz MI Ahmad A De Zutter A Siddiquei MM Blanter M. Evaluation of proteoforms of the transmembrane chemokines CXCL16 and CX3CL1, their receptors, and their processing metalloproteinases ADAM10 and ADAM17 in proliferative diabetic retinopathy. Front In Immunol (2020) 11:601639. doi: 10.3389/fimmu.2020.601639 33552057 PMC7854927

[11] Bromberg-White JL Glazer L Downer R Furge K Boguslawski E Duesbery NS. Identification of VEGF-independent cytokines in proliferative diabetic retinopathy vitreous. Invest Ophthalmol Vis Sci (2013) 54(10):6472–80. doi: 10.1167/iovs.13-12518 24003089

[12] Mason RH Minaker SA Lahaie Luna G Bapat P Farahvash A Garg A. Changes in aqueous and vitreous inflammatory cytokine levels in proliferative diabetic retinopathy: a systematic review and meta-analysis. Eye (Lond) (2022). doi: 10.1038/s41433-022-02127-x 35672457

[13] Whitehead M Wickremasinghe S Osborne A Van Wijngaarden P Martin KR. Diabetic retinopathy: a complex pathophysiology requiring novel therapeutic strategies. Expert Opin On Biol Ther (2018) 18(12):1257–70. doi: 10.1080/14712598.2018.1545836 PMC6299358 30408422

[14] Fahey E Doyle SL. IL-1 family cytokine regulation of vascular permeability and angiogenesis. Front In Immunol (2019) 10:1426. doi: 10.3389/fimmu.2019.01426 31293586 PMC6603210

[15] Simo-Servat O Simo R Hernandez C. Circulating biomarkers of diabetic retinopathy: An overview based on physiopathology. J Diabetes Res (2016) 2016:5263798. doi: 10.1155/2016/5263798 27376090 PMC4916280

[16] Smith GD. Mendelian randomization for strengthening causal inference in observational studies: Application to gene x environment interactions. Perspect Psychol Sci (2010) 5(5):527–45. doi: 10.1177/1745691610383505 26162196

[17] Swerdlow DI Kuchenbaecker KB Shah S Sofat R Holmes MV White J. Selecting instruments for mendelian randomization in the wake of genome-wide association studies. Int J Epidemiol (2016) 45(5):1600–16. doi: 10.1093/ije/dyw088 PMC5100611 27342221

[18] Han X Ong J-S An J Hewitt AW Gharahkhani P MacGregor S. Using mendelian randomization to evaluate the causal relationship between serum c-reactive protein levels and age-related macular degeneration. Eur J Epidemiol (2020) 35(2):139–46. doi: 10.1007/s10654-019-00598-z 31900758

[19] Richmond RC Davey Smith G Ness AR den Hoed M McMahon G Timpson NJ. Assessing causality in the association between child adiposity and physical activity levels: a mendelian randomization analysis. PloS Med (2014) 11(3):e1001618. doi: 10.1371/journal.pmed.1001618 24642734 PMC3958348

[20] Wang Q Shi Q Lu J Wang Z Hou J. Causal relationships between inflammatory factors and multiple myeloma: A bidirectional mendelian randomization study. Int J Cancer (2022) 151(10):1750–9. doi: 10.1002/ijc.34214 35841389

[21] Ahola-Olli AV Wurtz P Havulinna AS Aalto K Pitkanen N Lehtimaki T. Genome-wide association study identifies 27 loci influencing concentrations of circulating cytokines and growth factors. Am J Hum Genet (2017) 100(1):40–50. doi: 10.1016/j.ajhg.2016.11.007 27989323 PMC5223028

[22] Kurki MI Karjalainen J Palta P Sipilä TP Kristiansson K Donner K. FinnGen: Unique genetic insights from combining isolated population and national health register data. medRxiv (2022) medRxiv 2022.03.03 :22271360. doi: https://doi.org/10.1101/2022.03.03.22271360

[23] Pollack S Igo RP Jensen RA Christiansen M Li X Cheng C-Y. Multiethnic genome-wide association study of diabetic retinopathy using liability threshold modeling of duration of diabetes and glycemic control. Diabetes (2019) 68(2):441–56. doi: 10.2337/db18-0567 PMC6341299 30487263

[24] Burgess S Davies NM Thompson SG. Bias due to participant overlap in two-sample mendelian randomization. Genet Epidemiol (2016) 40(7):597–608. doi: 10.1002/gepi.21998 27625185 PMC5082560

[25] Early Treatment Diabetic Retinopathy Study Research Group. Grading diabetic retinopathy from stereoscopic color fundus photographs–an extension of the modified airlie house classification. ETDRS report number 10. Ophthalmology (1991) 98(5Suppl.):786–806. doi: 10.1016/S0161-6420(13)38012-9 2062513

[26] Burgess S Butterworth A Thompson SG. Mendelian randomization analysis with multiple genetic variants using summarized data. Genet Epidemiol (2013) 37(7):658–65. doi: 10.1002/gepi.21758 PMC4377079 24114802

[27] Forgetta V Manousaki D Istomine R Ross S Tessier M-C Marchand L. Rare genetic variants of Large effect influence risk of type 1 diabetes. Diabetes (2020) 69(4):784–95. doi: 10.2337/db19-0831 PMC7085253 32005708

[28] Mahajan A Taliun D Thurner M Robertson NR Torres JM Rayner NW. Fine-mapping type 2 diabetes loci to single-variant resolution using high-density imputation and islet-specific epigenome maps. Nat Genet (2018) 50(11):1505–13. doi: 10.1038/s41588-018-0241-6 PMC6287706 30297969

[29] Chen J Spracklen CN Marenne G Varshney A Corbin LJ Luan J.a.. The trans-ancestral genomic architecture of glycemic traits. Nat Genet (2021) 53(6):840–60. doi: 10.1038/s41588-021-00852-9 PMC7610958 34059833

[30] Evangelou E Warren HR Mosen-Ansorena D Mifsud B Pazoki R Gao H. Genetic analysis of over 1 million people identifies 535 new loci associated with blood pressure traits. Nat Genet (2018) 50(10):1412–25. doi: 10.1038/s41588-018-0205-x PMC6284793 30224653

[31] Yengo L Sidorenko J Kemper KE Zheng Z Wood AR Weedon MN. Meta-analysis of genome-wide association studies for height and body mass index in ∼700000 individuals of European ancestry. Hum Mol Genet (2018) 27(20):3641–9. doi: 10.1093/hmg/ddy271 PMC6488973 30124842

[32] Bowden J Holmes MV. Meta-analysis and mendelian randomization: A review. Res Synthesis Methods (2019) 10(4):486–96. doi: 10.1002/jrsm.1346 PMC6973275 30861319

[33] Machiela MJ Chanock SJ. LDlink: a web-based application for exploring population-specific haplotype structure and linking correlated alleles of possible functional variants. Bioinformatics (2015) 31(21):3555–7. doi: 10.1093/bioinformatics/btv402 PMC4626747 26139635

[34] Hartwig FP Davies NM Hemani G Davey Smith G. Two-sample mendelian randomization: avoiding the downsides of a powerful, widely applicable but potentially fallible technique. Int J Epidemiol (2016) 45(6):1717–26. doi: 10.1093/ije/dyx028 PMC5722032 28338968

[35] Bowden J Del Greco M F Minelli C Davey Smith G Sheehan N Thompson J. A framework for the investigation of pleiotropy in two-sample summary data mendelian randomization. Stat In Med (2017) 36(11):1783–802. doi: 10.1002/sim.7221 PMC5434863 28114746

[36] Burgess S Thompson SG. Interpreting findings from mendelian randomization using the MR-egger method. Eur J Epidemiol (2017) 32(5):377–89. doi: 10.1007/s10654-017-0255-x PMC5506233 28527048

[37] Bowden J Smith GD Haycock PC Burgess S. Consistent estimation in mendelian randomization with some invalid instruments using a weighted median estimator. Genet Epidemiol (2016) 40(4):304–14. doi: 10.1002/gepi.21965 PMC4849733 27061298

[38] Verbanck M Chen CY Neale B Do R. Detection of widespread horizontal pleiotropy in causal relationships inferred from mendelian randomization between complex traits and diseases (vol 50, 693, 2018). Nat Genet (2018) 50(8):1196–6. doi: 10.1038/s41588-018-0164-2 PMC6083837 29686387

[39] Hemani G Tilling K Davey Smith G. Orienting the causal relationship between imprecisely measured traits using GWAS summary data. PloS Genet (2017) 13(11):e1007081. doi: 10.1371/journal.pgen.1007081 29149188 PMC5711033

[40] Ibrahim AS El-Remessy AB Matragoon S Zhang W Patel Y Khan S. Retinal microglial activation and inflammation induced by amadori-glycated albumin in a rat model of diabetes. Diabetes (2011) 60(4):1122–33. doi: 10.2337/db10-1160 PMC3064086 21317295

[41] Vujosevic S Simo R. Local and systemic inflammatory biomarkers of diabetic retinopathy: An integrative approach. Invest Ophthalmol Visual Sci (2017) 58(6):Bio68–75. doi: 10.1167/iovs.17-21769 28510630

[42] Petrovic MG Korosec P Kosnik M Hawlina M. Association of preoperative vitreous IL-8 and VEGF levels with visual acuity after vitrectomy in proliferative diabetic retinopathy. Acta Ophthalmol (2010) 88(8):e311–316. doi: 10.1111/j.1755-3768.2010.02030.x 21073666

[43] MacKinnon JR Knott RM Forrester JV. Altered l-selectin expression in lymphocytes and increased adhesion to endothelium in patients with diabetic retinopathy. Br J Ophthalmol (2004) 88(9):1137–41. doi: 10.1136/bjo.2003.040329 PMC1772318 15317703

[44] Loporchio DF Tam EK Cho J Chung J Jun GR Xia W. Cytokine levels in human vitreous in proliferative diabetic retinopathy. Cells (2021) 10(5):1069. doi: 10.3390/cells10051069 PMC8147162 33946446

[45] Teijeira A Garasa S Ochoa MC Villalba M Olivera I Cirella A. IL8, neutrophils, and NETs in a collusion against cancer immunity and immunotherapy. Clin Cancer Res (2021) 27(9):2383–93. doi: 10.1158/1078-0432.CCR-20-1319 33376096

[46] Raghuwanshi SK Su Y Singh V Haynes K Richmond A Richardson RM. The chemokine receptors CXCR1 and CXCR2 couple to distinct G protein-coupled receptor kinases to mediate and regulate leukocyte functions. J Immunol (2012) 189(6):2824–32. doi: 10.4049/jimmunol.1201114 PMC3436986 22869904

[47] Sheemar A Soni D Takkar B Basu S Venkatesh P. Inflammatory mediators in diabetic retinopathy: Deriving clinicopathological correlations for potential targeted therapy. Indian J Ophthalmol (2021) 69(11):3035–49. doi: 10.4103/ijo.IJO_1326_21 PMC8725076 34708739

[48] Schoenberger SD Kim SJ Shah R Sheng J Cherney E. Reduction of interleukin 8 and platelet-derived growth factor levels by topical ketorolac, 0.45%, in patients with diabetic retinopathy. JAMA Ophthalmol (2014) 132(1):32–7. doi: 10.1001/jamaophthalmol.2013.6203 24336915

[49] Sukowati CHC Patti R Pascut D Ladju RB Tarchi P Zanotta N. Serum stem cell growth factor beta for the prediction of therapy response in hepatocellular carcinoma. BioMed Res Int (2018) 2018:6435482. doi: 10.1155/2018/6435482 30246025 PMC6136561

[50] Hiraoka A Sugimura A Seki T Nagasawa T Ohta N Shimonishi M. Cloning, expression, and characterization of a cDNA encoding a novel human growth factor for primitive hematopoietic progenitor cells. Proc Natl Acad Sci U.S.A. (1997) 94(14):7577–82. doi: 10.1073/pnas.94.14.7577 PMC23864 9207134

[51] Balogh A Milibak T Szabo V Nagy ZZ Kaarniranta K Resch MD. Immunological biomarkers of the vitreous responsible for proliferative alteration in the different forms of retinal detachment. BMC Ophthalmol (2020) 20(1):491. doi: 10.1186/s12886-020-01745-x 33371882 PMC7768644

[52] Duh EJ Sun JK Stitt AW. Diabetic retinopathy: current understanding, mechanisms, and treatment strategies. JCI Insight (2017) 2(14):e93751. doi: 10.1172/jci.insight.93751 28724805 PMC5518557

[53] Rubsam A Parikh S Fort PE. Role of inflammation in diabetic retinopathy. Int J Mol Sci (2018) 19(4):942. doi: 10.3390/ijms19040942 PMC5979417 29565290

[54] Butler JM Guthrie SM Koc M Afzal A Caballero S Brooks HL. SDF-1 is both necessary and sufficient to promote proliferative retinopathy. J Clin Invest (2005) 115(1):86–93. doi: 10.1172/JCI22869 15630447 PMC539202

[55] Lange CAK Stavrakas P Luhmann UFO de Silva DJ Ali RR Gregor ZJ. Intraocular oxygen distribution in advanced proliferative diabetic retinopathy. Am J Ophthalmol (2011) 152(3):406–412.e403. doi: 10.1016/j.ajo.2011.02.014 21723532

[56] Ludwig PE Freeman SC Janot AC. Novel stem cell and gene therapy in diabetic retinopathy, age related macular degeneration, and retinitis pigmentosa. Int J Retina Vitreous (2019) 5:7. doi: 10.1186/s40942-019-0158-y 30805203 PMC6373096

[57] Sun H Zou W Zhang Z Huang D Zhao J Qin B. Vitreous inflammatory cytokines and chemokines, not altered after preoperative adjunctive conbercept injection, but associated with early postoperative macular edema in patients with proliferative diabetic retinopathy. Front Physiol (2022) 13:846003. doi: 10.3389/fphys.2022.846003 35309074 PMC8928061

[58] Rangasamy S McGuire PG Franco Nitta C Monickaraj F Oruganti SR Das A. Chemokine mediated monocyte trafficking into the retina: role of inflammation in alteration of the blood-retinal barrier in diabetic retinopathy. PloS One (2014) 9(10):e108508. doi: 10.1371/journal.pone.0108508 25329075 PMC4203688

[59] Keindl M Fedotkina O du Plessis E Jain R Bergum B Mygind Jensen T. Increased plasma soluble interleukin-2 receptor alpha levels in patients with long-term type 1 diabetes with vascular complications associated with IL2RA and PTPN2 gene polymorphisms. Front Endocrinol (Lausanne) (2020) 11:575469. doi: 10.3389/fendo.2020.575469 33193091 PMC7664831

