# Supplementary material for: Systemic inflammatory regulators and proliferative diabetic retinopathy: A bidirectional Mendelian randomization study
Source: Front Immunol. 2023 Feb 10;14:1088778. doi: 10.3389/fimmu.2023.1088778 (PMC9950638; doi:10.3389/fimmu.2023.1088778)
Supplement: Supplementary file 1 [file DataSheet_1.docx]

**
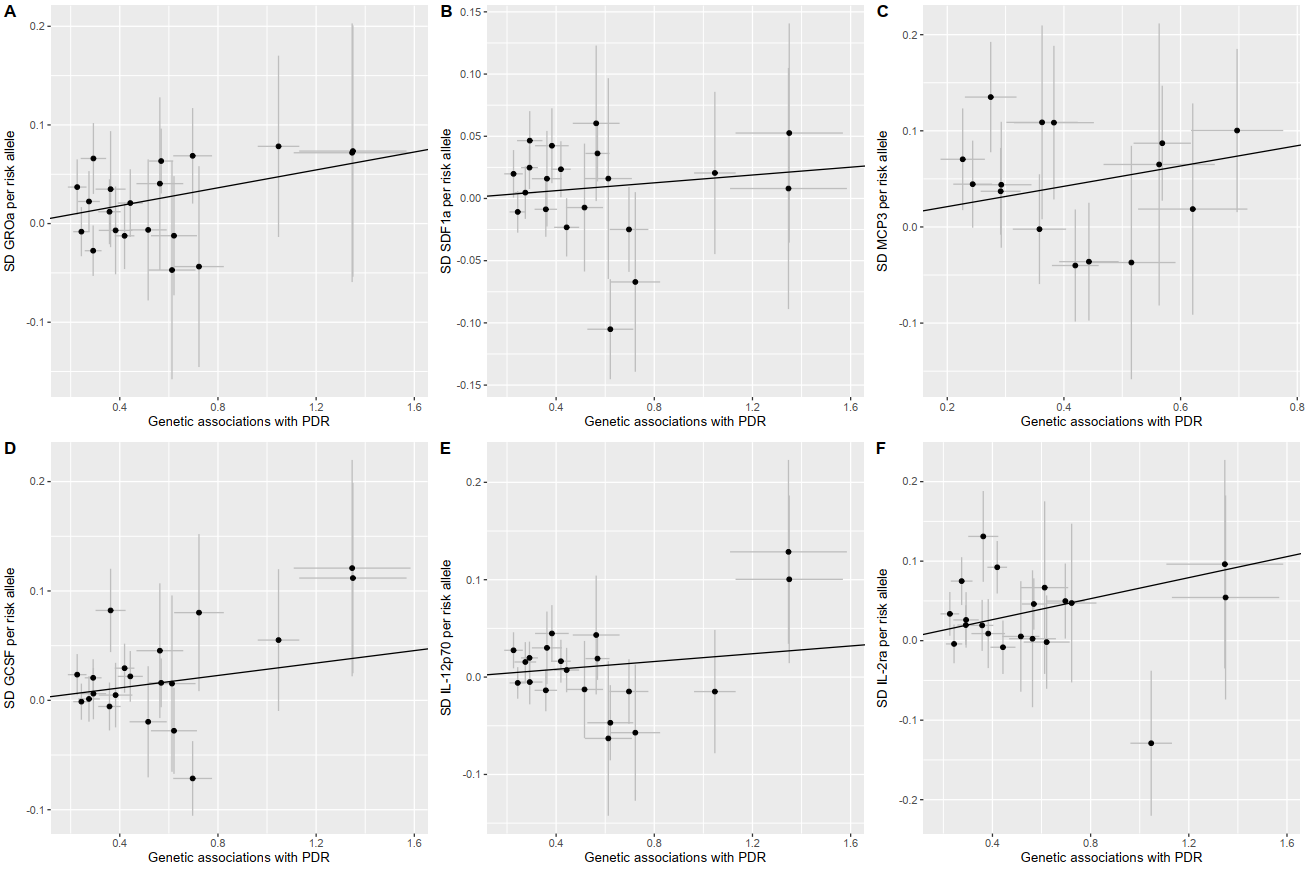
**

**Figure S1**. Scatter plot of PDR related SNPs with inflammatory regulators, with summary-level data on PDR from FinnGen.

A) Genetic association of PDR with GROa. B) Genetic association of PDR with SDF1a. C) Genetic association of PDR with MCP3. D) Genetic association of PDR with GCSF. E) Genetic association of PDR with IL-12p70. F) Genetic association of PDR with IL-2ra. Black line indicates the estimate of effect using the inverse variance weighted method. Error bars indicate 95% confidence intervals.

Abbreviations: GCSF, granulocyte colony-stimulating factor; GROa, growth-regulated oncogene-alpha; IL, interleukin; MCP3, monocyte chemotactic protein-3; PDR, proliferative diabetic retinopathy; SD, standard deviation; SDF1a, stromal cell-derived factor 1.

**
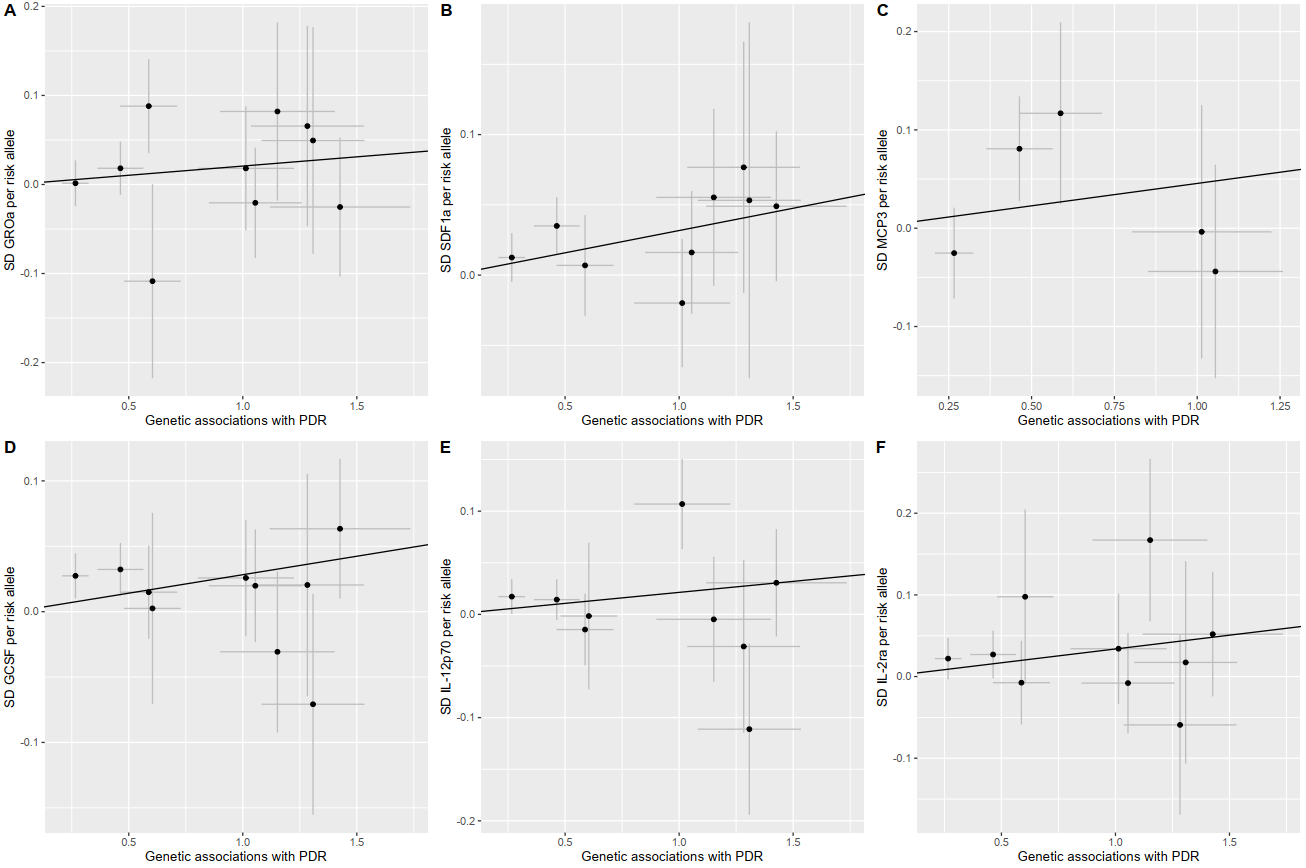
**

**Figure S2**. Scatter plot of PDR related SNPs with inflammatory regulators, with summary-level data on PDR from eight cohorts.

A) Genetic association of PDR with GROa. B) Genetic association of PDR with SDF1a. C) Genetic association of PDR with MCP3. D) Genetic association of PDR with GCSF. E) Genetic association of PDR with IL-12p70. F) Genetic association of PDR with IL-2ra. Black line indicates the estimate of effect using the inverse variance weighted method. Error bars indicate 95% confidence intervals.

Abbreviations: GCSF, granulocyte colony-stimulating factor; GROa, growth-regulated oncogene-alpha; IL, interleukin; MCP3, monocyte chemotactic protein-3; PDR, proliferative diabetic retinopathy; SD, standard deviation; SDF1a, stromal cell-derived factor 1.
